# Attitudes toward medicalization in childbirth and their relationship with locus of control and coping in a Spanish population

**DOI:** 10.1186/s12884-022-04748-2

**Published:** 2022-06-28

**Authors:** Maite Espinosa, Isabel Artieta-Pinedo, Carmen Paz-Pascual, Paola Bully-Garay, Arturo García-Álvarez, ema-Q. Group, Itziar Estalella, Mª José Trincado, Inés Cabeza, Mari Pierre Gagnon, Ana Fernández, Gorane Lozano, Gemma Villanueva, Jesús Sánchez, Amaia Maquibar, David Moreno, Catalina Legarra, Maria Jesús Mulas, Mónica Blas, Pilar Amorrortu, Sonia Alva

**Affiliations:** 1 Osakidetza-Basque Health Service, Biocruces-Bizkaia Health Research Institute, C/ Edificio Biocruces 3, Plaza De Cruces, 48903 Barakaldo, Spain; 2 Primary Care Midwife Zuazo Health Centre, OSI BARAKALDO-SESTAO-OSAKIDETZA, C/ Lurkizaga Kalea, s/n, 48902 Barakaldo, Spain; 3 Associate Professor of the School of Nursing, University of the Basque Country, C/ Barrio Sarriena S/N, 48940 Leioa, Spain; 4 Primary Care Midwife Markonzaga Health Centre, OSI BARAKALDO-SESTAO-OSAKIDETZA, C/ Antonio Trueba Kalea 17, 48910 Sestao, Spain; 5 Lecturer in the Midwifery Training Unit of the Basque Country, Hospital de Basurto-OSAKIDETZA, C/ Montevideo Etorbidea 18, 48013 Bilbao, Spain; 6 Paola Bully Methodological and Statistical Consultant, C/ Barrio La Sota, 48190 Sopuerta, Spain; 7 Osakidetza, University of Basque Country, University of Laval, Biocruces Bizkaia Health Research Institute, C/ Edificio Biocruces 3, Plaza De Cruces, 48903 Barakaldo, Spain

**Keywords:** Attitude towards medicalization of childbirth, Locus of control, Coping, Pregnancy

## Abstract

The dominant model of childbirth in most Western countries is medicalized childbirth. Women's beliefs about whether childbirth should be a medicalized process to a greater or lesser degree may be related, in addition to contextual factors, to internal factors. The objective of the study is to find out if women’s locus of control (LC) and stress coping strategies (CS) are related to having a more favourable or less favourable attitude towards medicalization (ATMC). A cross-sectional study was carried out with the participation of 248 women recruited in primary care centres by their midwives. All the women filled in answers on a mobile phone app with various different measurement instruments: the questionnaire created by Benyamini to evaluate their ATMC; the Spanish version of the Wallston MLC to evaluate their LC; and the Spanish adaptation of the “Revised Prenatal Coping Inventory (NuPCI)” scale for the assessment of their CS. The women presented a favourable attitude towards medicalization, with a mean ATMC score of 3.42. Both the LC and the CS of women during pregnancy are related to this attitude. Specifically, having an internal LC and using preparative CS both lower the probability of presenting a favourable attitude towards medicalization, while the lack of a paid job raises the probability. For each point in internal locus and preparatory coping, the ATMC score decreased by 0.02 and 0.23 points, respectively, while it increased by 0.18 for not having a paid job. The influence of these psychological factors must be taken into account in the development of content and interventions that promote a more natural birth.

## Background

The dominant model of childbirth in most Western countries is medicalized childbirth [1]. In 2007, the Spanish Ministry of Health and Consumer Affairs drew up the “Strategy for care of normal childbirth in the National Health System”. With broad institutional participation, scientific societies, experts, citizens’ associations and representatives of the Autonomous Communities, the aim was offering multidisciplinary care to women, newborns and families during labour and birth, guaranteeing adequate conditions of safety, quality and efficiency in the management of hospital maternity. For more than a decade, the National Sexual and Reproductive Health Strategy of the Spanish NHS (ENSSR) has been promoting a model of care for childbirth, based on scientific evidence and, therefore, attentive to the needs of the labouring woman and the newborn human being (NB), respectful of the physiology of labour and birth, advocating the least possible interventionism, the personalisation of care, as well as the principle of women's autonomy. In 2018, of the total number of births attended, 81.1% took place in the NHS. With regard to the type of delivery, while in 2018 NHS network hospitals had 21.8% of deliveries by caesarean section, in private hospitals this rate was 36.5%. Caesarean section rates persist above the World Health Organisation (WHO) reference standards (less than 15%). The percentage of amniotomy was 13% and the percentage of induced deliveries was 34.2%, compared to the WHO recommendation of 10%. Epidural anaesthesia was used in 62.9% of deliveries, an episiotomy was performed in 27.5%, and 17.5% of deliveries were instrumental. These data are some of the conclusions of the descriptive analysis of the data related to perinatal care in the period 2010–2018, focusing on hospital health services. The sources of information are the databases of the Specialised Care Information Systems (SIAE), Minimum Basic Data Set (CMBD) (2010–2015) and Specialised Healthcare Activity Register (RAE-CMBD) (2016–2018) [2].

Medicalization begins at pregnancy, with prenatal care that transforms pregnancy into a condition of permanent risk, which requires medical monitoring [3]. Although medical and technological advances in maternity care have drastically reduced maternal and infant mortality, the danger of introducing the routine use of technology in birth is that it can cause what has been called in the literature ‘a cascade of intervention’, where one intervention leads to another [4].

The concept of medicalization emerged in the 1970s, and most of the literature on this topic still comes from the social sciences [5–7], although there are some authors in medical academia who address this topic [8, 9]. Kishore’s *Dictionary of Public Health* [10] defines medicalization as “the way in which the field of modern medicine encompasses many problems that were not previously considered medical entities”, and adds that it includes a wide variety of manifestations, such as the normal phases of the reproductive and vital cycle of women (menstruation, pregnancy, childbirth and menopause), among others.

The medicalization of pregnancy and childbirth benefited, in its genesis, from the emergence of biomedicine and evidence-based medicine, the development of obstetrics and the generalization of surgery, and the development of the preventive approach and the explosion of new technologies applied to biomedicine. The generalization of the phenomenon was favoured by economic, political, social and cultural factors [11–13].

Health professionals necessarily function as agents of this medicalization, but we should consider their situation with respect to social expectations and demands [11] and review in what way a greater participation by patients in decision-making would influence the delivery process. It could be the case that patients contribute to medicalization through their “desire and demand”- in relation to both contextual factors (advertising, media, social networks and the Internet), and psychological variables [13–17]- or on the contrary, that they are influenced by the numerous initiatives that advocate a more natural birth or greater control of the process [18, 19]. This last choice would mean distancing themselves from the dominant medical model [20, 21] and assuming responsibility for the outcome of the delivery [22].

The fact that a woman has a more favourable or less favourable attitude towards medicalization will influence the choices she will make in relation to labour, the degree of planning for it, and finally the outcome and experience of childbirth [22–24]; hence it is important to know what factors, in addition to contextual ones, influence or are related to attitudes towards medicalization. Benyamini et al. [1] found that, in Israel, the attitude was more favourable among young women, with less education, who were immigrants or had a complicated obstetric history. These attitudes, measured during pregnancy, were related to a greater fear of childbirth and a greater probability of having a more medicalized delivery. Continuing with the factors related to or underlying these attitudes or beliefs and the choices they make, a cohort of Swedish and Australian women were categorized into three attitudinal profiles:*’Self determiners’, ‘Take it as it comes’* and *‘Fearful’* [24]. Comparison between these groups revealed that the “fearful” group was more likely to have poorer self-assessed emotional health than the “self determiners” group, to prefer a caesarean section, to show less positive feelings towards the pregnancy, and to choose an epidural delivery etc.; the “take it as it comes” group was more likely to accept obstetric interventions when expressed as being for the well-being of the child, and the *“self determiners”* group was more inclined towards a more natural birth and assuming greater control over it. These three attitudinal profiles described by Haines could be related to different types of locus.

The locus of control (LOC), which was first used by Rotter in social learning theory (SLT) and served as the basis for the Wallston scale [25, 26], is one of the concepts used in understanding health behaviours and beliefs. This concept is defined as the degree to which people believe their health is controlled by internal or external factors [26]. According to this idea, individuals can be classified as internal or external. People with internal LOC are convinced that they can control their own destiny, they feel responsible for their lives and their behaviour, and they are capable of internal growth and improvement through effort and the development of their abilities. These people assume that their health results are a function of the behaviours they have, and they use the information provided to improve and take control of their own health [27–31]. On the contrary, the external LOC is typical of those individuals with a tendency to consider that their health outcomes, or what happens to them, is under the control of other people, or is predetermined, or uncontrollable, since it depends on external factors outside their control, such as luck, destiny, chance or the supernatural [32]. The locus of control has been seen to influence the choices women make at the time of childbirth; for example, a woman with internal locus of control, or external luck, is more likely to attempt a vaginal birth after a caesarean section than a woman with an external locus of control—powerful others [33]. The identification of the type of locus of control has proven to be useful, therefore, for predicting trends in health behaviours and could predict what type of attitude towards childbirth –medicalized to a greater or lesser degree– the woman will have.

Another important factor to consider is the evaluation that the woman makes of the future childbirth as a potentially stressful event, and the coping strategies that she is going to use in that situation. *Stress* can be defined as a demand appraised as taxing or exceeding the resources of the individual [34]. Coping styles are the stable tendencies to respond to the stressing event with a certain type of strategy, and strategies are specific ways to cope with stress. Some authors describe 3 types of coping strategies: 1) coping considered positive, based on preparation, planning strategies and focuses on the problem to be solved; 2) a style, in this case negative or of avoidance, focused on the emotion generated and used to escape from the distressing situation; and 3) a “spiritual” style that faces the situation by trusting in a higher power [35–37]. Knowing if the type of coping strategies used by women during pregnancy are related to having a more favourable or less favourable attitude towards medicalization is of interest, so that this variable can be taken into account in the development of interventions. For example, if it is seen that the use of preparatory strategies can influence having an unfavourable attitude towards a medicalized delivery, use of such strategies could be encouraged during pregnancy. Moreover, if we see that adopting an avoidance strategy during pregnancy is related to medicalization, we will have to be alert and work on the use of other more adaptive strategies.

The objective of this paper is therefore to evaluate whether women’s locus of control and stress coping strategies are related to having a more favourable or less favourable attitude towards medicalization in childbirth, and if so, what type of locus of control and what type of coping strategy would be more related.

## Methods

### Design and selection of participants

The study data is part of a larger investigation into the development of two instruments designed to detect the needs of women during pregnancy, childbirth and postpartum. The study protocol has been published previously [38].

This is a cross-sectional study, collecting data in the Basque Health Service between 2019 and early 2020. The Osakidetza-Basque Health Service is the public healthcare system of the Basque Country, a region located in the north of Spain with a population of just over two million inhabitants. Osakidetza was created by the Health Department of the Basque Government in 1983, and all the public hospitals and primary care centres of the Basque region come under this organization, structured into 13 Integrated Health Organizations (IHO) spread throughout the Basque country. An OSI is a network consisting of several primary care centres, dispensaries for sparsely populated rural areas, and a referral hospital. More than 30,000 professionals work for Osakidetza, which could be considered the largest health organization in the Basque Country. For pregnancy, delivery and postpartum follow-up, each hospital coordinates with a set of primary care centres. On average, one midwife works in each primary care health centre.

The women were recruited from primary care centres, under the management of several IHOs, by their midwives during a pregnancy check-up, and also through snowball sampling. The midwife informed women over 18 years of age with a good knowledge of the Spanish about the study and, if the women wished to participate, the research team provided them with a link to the questionnaires on their mobile phone. The profile of the woman who goes to the midwife for pregnancy control is that of a woman whose pregnancy is low risk, as high-risk pregnancies are supervised by the obstetrician. Additionally, the women were able to share the link with other pregnant women. In the same app, an informed consent request was made which, once accepted, gave access to the questionnaires (the measurement instruments and a formulary of sociodemographic and clinical questions). The study was approved by the Basque Clinical Research Ethics Committee (PI2019110).

### Measurement instruments

To assess Attitudes towards the Medicalization of Childbirth (ATMC) among pregnant women, a questionnaire of the same name created by Benyamini et al. [1] was used. The questionnaire consists of 15 items aimed at measuring women’s inclination to use medical technology and interventions during childbirth. Seven items expressed a positive ATMC and eight items expressed a negative attitude, and women were asked to rate their level of agreement with each statement on 1–5 Likert scales. The negative items were reversed and then a summary score was calculated as the average of all responses. A high score indicates a positive attitude towards medicalization.

To assess the locus of control style of the participants, the Spanish version of the Wallston Form ‘A Multidimensional Locus of Control’ was used, adapted by Tomás-Sábado and Montes-Hidalgo in 2016 [32]. It consists of 18 items, with 6 response possibilities for each of them, scoring from 1 = completely agree, to 6 = completely disagree. The scale provides 3 relatively independent scores corresponding to the 3 factors considered: *internality* (items 1, 6, 8, 12, 13 and 17), which expresses beliefs that health depends on one’s own behaviour; and *chance/luck* (2, 4, 9, 11, 15 and 16) and *other relevant people* [3, 5, 7, 10, 14, 18] which both express beliefs in externality, that is, they consider that health depends on chance/luck or on the actions of other competent people, respectively.

To evaluate the coping strategies of the women, the Spanish adaptation by Lorén-Guerrero [37] of the scale called “Revised Prenatal Coping Inventory (NuPCI)” developed for the assessment of coping strategies over the course of pregnancy [36] was used. The scale consists of 42 items. Women are asked to report how often they used each method of coping “to try to manage the strains and challenges of being pregnant” during a given time frame on a scale ranging from 0 (never) to 4 (almost always). The items refer to general coping strategies along with others that are specific to pregnancy, and are divided into 3 scales or types of coping: a) the preparatory scale, with 15 questions [37], b) the avoidant coping scale, with 11 questions and c) the spiritual coping scale, with 6 questions.

Fear of childbirth was also evaluated, since some studies relate it to ATMC, using a Spanish version of Wijma’s 33-item questionnaire from 2005 [39], adapted by Ortega-Cejas et al. [40], which maintained the one-dimensional structure proposed by the author of the original test. The answers to each question appear as a 6-point Likert scale, the answers being (0 and 5 respectively) the opposite extremes of a certain feeling or thought when the woman imagines her labour and delivery. The minimum score is 0 and the maximum is 165. Scores above 85 indicate severe fear of childbirth [40, 41].

The possible confounding effect and modifier of the effect of other variables that could be related to both ATMC and the locus of control and coping have been considered. These variables were collected through an introductory form, composed of 22 items, on sociodemographic and clinical data completed in the app: age, parity, nationality (Spanish/immigrant), educational level (low/middle /high), paid work (yes/no) and the presence of certain prior risk factors (such as obesity, toxic habits, high age, history of prematurity or low birth weight, high birth weight, previous miscarriage or stillbirth, illness requiring periodic medical monitoring, regular medication, family history of births, etc.) with two possibilities of answering yes/no.

An analysis of the tests used was carried out to see if they presented adequate psychometric properties in our sample. All the tests used showed appropriate psychometric properties. In the case of ATMC, the original one-dimensional structure showed an appropriate adjustment in the adaptation made for our linguistic and cultural context (χ^2^ = 402.49, gl = 90, χ^2^/gl = 4.47, CFI = 0.94, TLI = 0.93, SRMR = 0.09). Internal reliability was high (omega index = 0.85, ordinal alpha = 0.80). In the Locus of Control Multidimensional Scale, the internality factor items formed a single dimension in our sample (χ^2^ = 28.92, df = 9, χ^2^/gl = 3.21, CFI = 0.98, TLI = 097, SRMR = 0.06) which presented acceptable internal consistency (omega = 0.75, ordinal alpha = 0.70). The chance/luck factor also showed a good fit between the one-dimensional solution and the data (χ^2^ = 42.26, df = 9, χ^2^/gl = 4.69, CFI = 0.98, TLI = 0.96, SRMR = 0.07) and presented high internal consistency (omega = 0.76, ordinal alpha = 0.75). The same was so with the factor “Other relevant people” (χ^2^ = 37.82, df = 9, χ^2^/gl = 4.20, CFI = 0.90, TLI = 0.91, SRMR = 0.08, omega = 0.75, ordinal alpha = 0.52). Regarding the NuPCI scale, a good fit was found in a) the preparatory coping scale (χ^2^ = 323.32, gl = 90, χ^2^/gl = 3.59, CFI = 0.95, TLI = 0.94, SRMR = 0.08, omega = 0.85, ordinal alpha = 0.85), b) the avoidant coping scale, (χ^2^ = 79.35, gl = 44, χ^2^/gl = 1.80, CFI = 0.97, TLI = 0.96, SRMR = 0.07, omega = 0.74, ordinal alpha = 0.79) and c) the spiritual coping scale, (χ^2^ = 9.76, gl = 9, χ^2^/gl = 1.08, CFI = 0.99, TLI = 0.99, SRMR = 0.05, omega = 0.81, ordinal alpha = 0.83). Finally, the W-DEC questionnaire also showed a good fit with our data (χ^2^ = 1315.53, df = 495, χ^2^/df = 2.66, CFI = 0.91, TLI = 0.90, SRMR = 0.11, omega = 0.92, ordinal alpha = 0.92.

### Statistical analysis

For the descriptive analyses, means and standard deviations were used for the continuous variables, and absolute and relative frequencies for the categorical variables.

For the analysis of the dimensional structure of the measurement instruments, confirmatory factor analyses were carried out and for the evaluation of their internal consistency, the McDonald's omega and ordinal alpha indices were calculated.

To analyse the relationship between the possible predictor variables (sociodemographic factors, locus, NuPCI, and fear) and ATMC, bivariate analyses were performed using linear regression for continuous variables and ANOVA for categorical variables. Multivariate ANCOVA models were built with all the predictor variables, obtaining the estimators and their 95% confidence intervals. The best model was chosen following a stepwise backward strategy using likelihood ratio tests (with a significance criteria of *P* < 0.05).

All analyses were performed with SAS 9.4 and R.

## Results

**Fig. 1:**
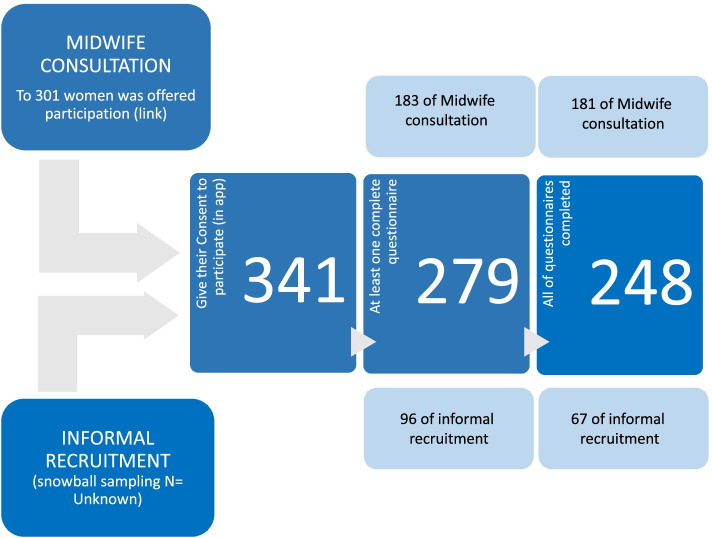
Flow of participants throughout the study

Participation in the study was offered to 301 women in 28 primary care centres, under the management of 6 IHO, by 25 midwives; in addition, the women were allowed to facilitate the link to other pregnant women. The number of women contacted by other women is not quantifiable, but we did know how many of them accessed the link and signed the consent form. Of the 341 women in total who accessed the mobile app and gave their consent to participate, 279 completed at least one questionnaire, and 248 of them completed all the questionnaires between weeks 8 and 41 of gestation (Fig. 1). Participants’ sociodemographic characteristics are presented in Table 1. Their mean age was 33.4 (± 4.74). They were recruited on average at 29.6 (± 8.0) weeks of gestation and 71.3% were primiparous.

**Table 1:**
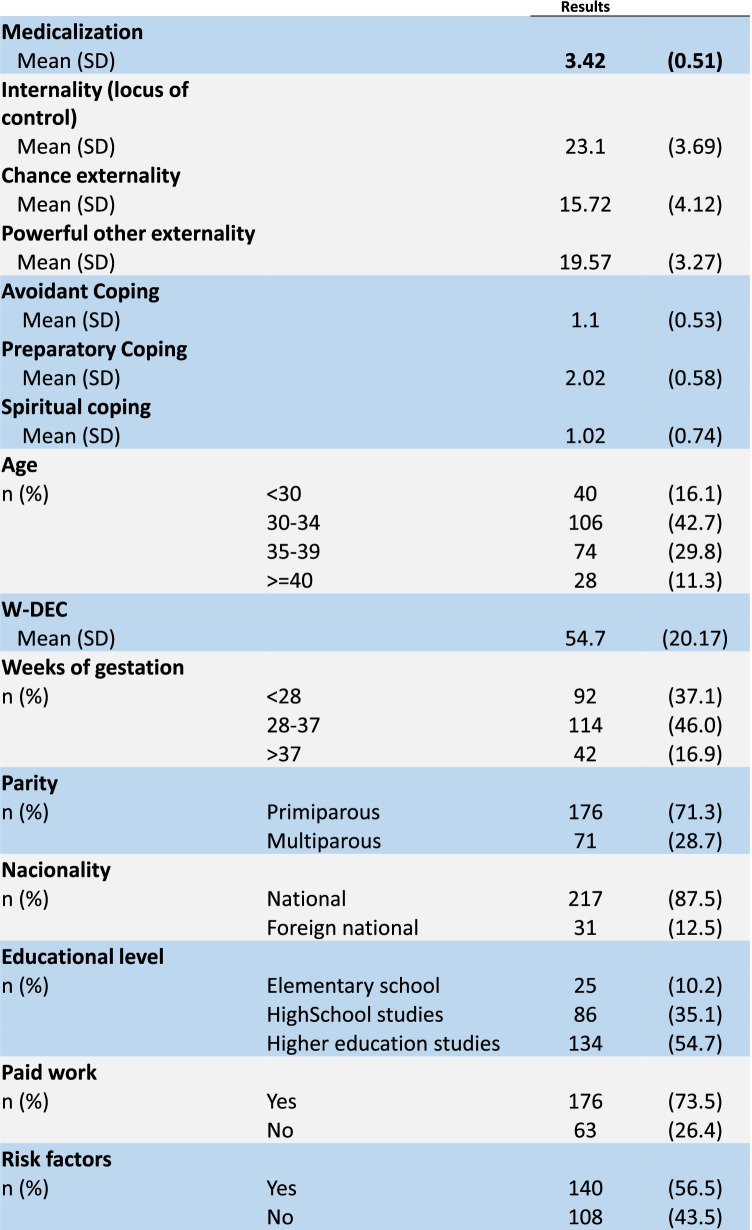
Scores obtained in attitude towards medicalization, locus of control, coping style and main characteristics of the sample

**Fig. 2:**
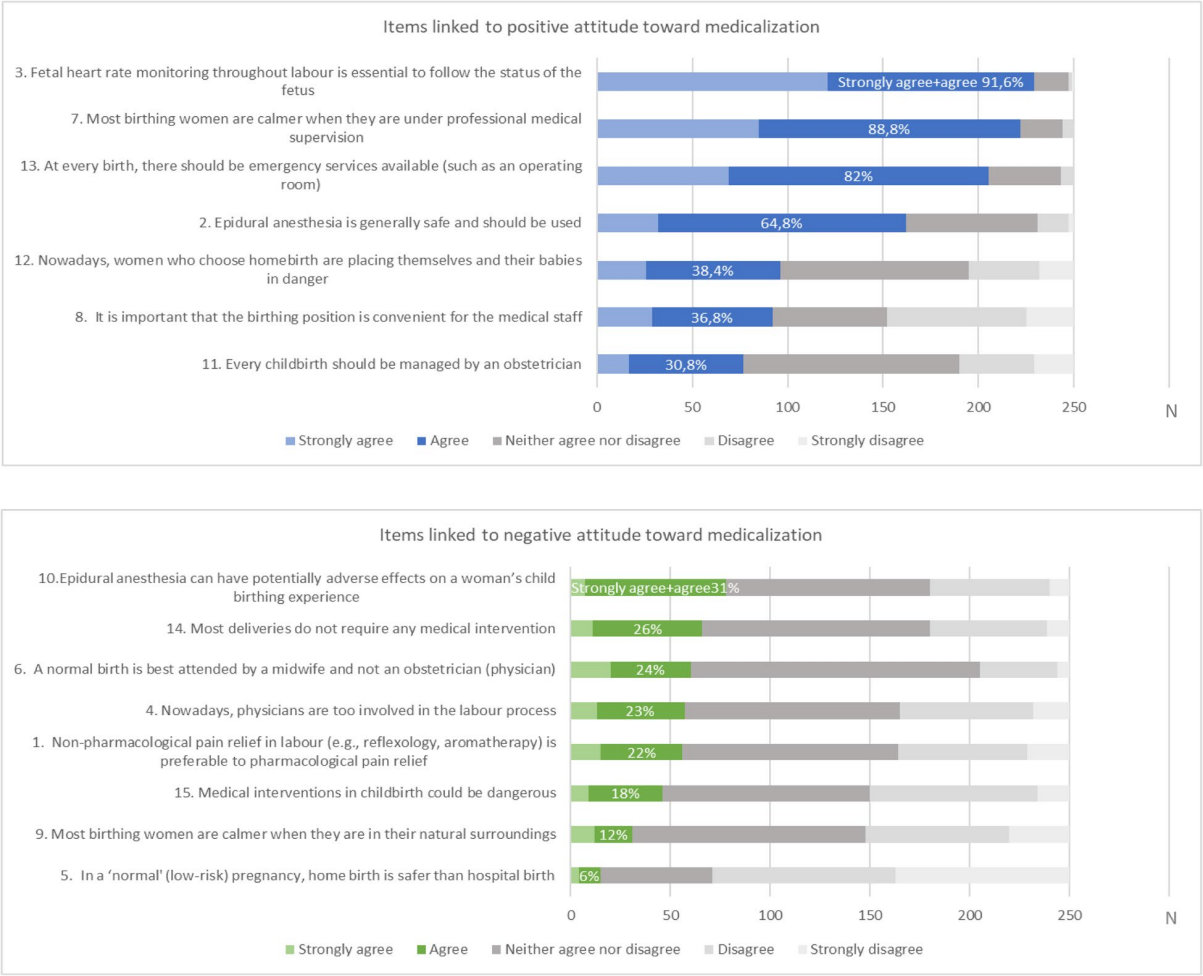
Medicalization of Childbirth questionnaire: *frequency and percentage of agreement on items linked to a positive attitude towards medicalization (above) vs agreement on items linked to a negative attitude towards medicalization (below). (N* = *250)*

The average ATMC score, as can be seen in Table 1, is 3.42; that is, favourable to medicalization in childbirth. In the present sample the mean scores ranged from 1.66 to 5 points. As in the original version of the questionnaire, no cut-off points were established for its use in our study. A summary score, after reversing negative items, was calculated as the mean of all responses. A high score indicates a positive attitude towards medicalization, with 1 being the lowest possible score and 5 the highest. Figure 2 shows the frequency of the items related to having a favourable attitude towards medicalization in childbirth vs. the frequency of items favourable to a less medicalized delivery. The issues that present a higher frequency of agreement (> 80%) are those about continuous foetal monitoring, medical supervision and the availability of emergency services; 64.8% consider the epidural a safe method and agree with its use. Other questions present a lower frequency of agreement (Fig. 2).

The mean score for the Internal locus of control was 23.1 (SD: 3.69); 15.72 (SD: 4.12) for Chance external locus; and 19.57 (SD: 3.27) for Powerful other externality. The mean score for the preparatory-scale questions of the Revised Prenatal Coping Inventory was 2.02 (SD: 0.58); 1.1 (SD: 0.53) for the avoidant scale; and 1.02 (SD: 0.74) for the spiritual scale. The mean score for W-DEC was 54.7 (SD: 20.17) (Table 1).

**Fig. 3:**
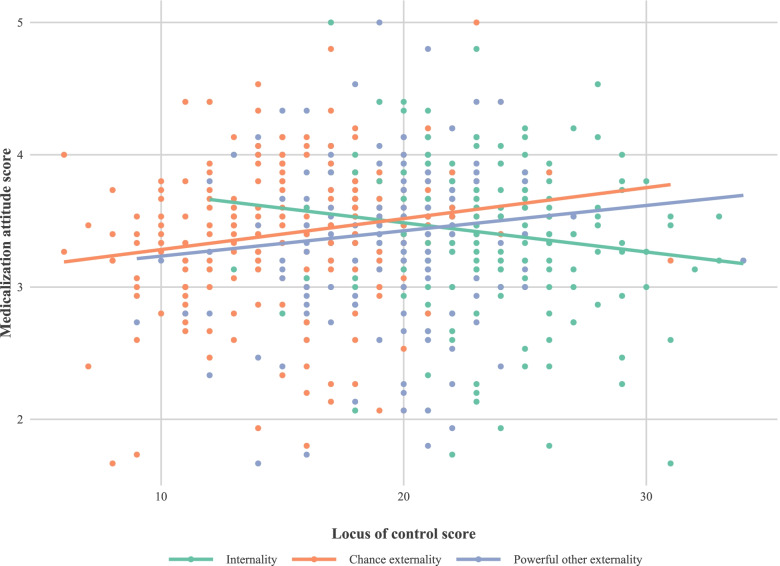
Relationship between the locus of control (“Internality”, “Chance externality” and “Powerful other externality” scores) and the score in attitude towards medicalization for each one

**Fig. 4:**
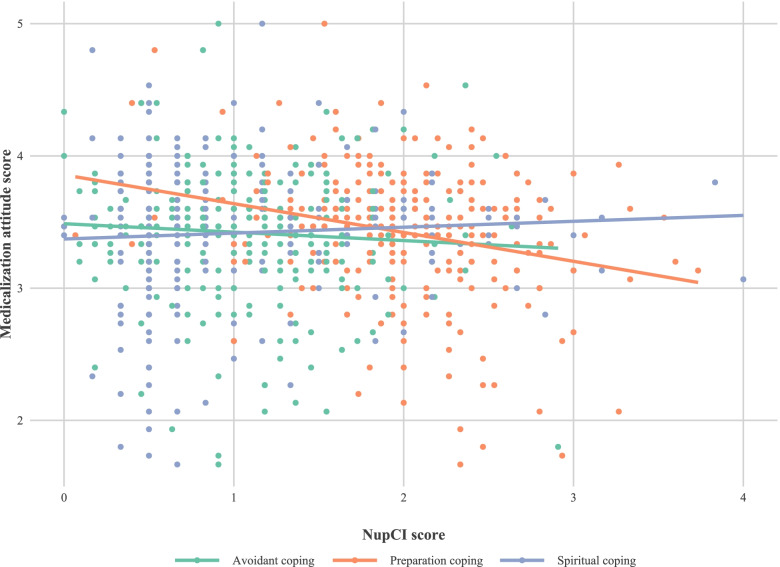
Relationship between the Coping style (“avoidant”, “preparatory” and “spiritual” scores) and the score in attitude towards medicalization for each one

**Fig. 5:**
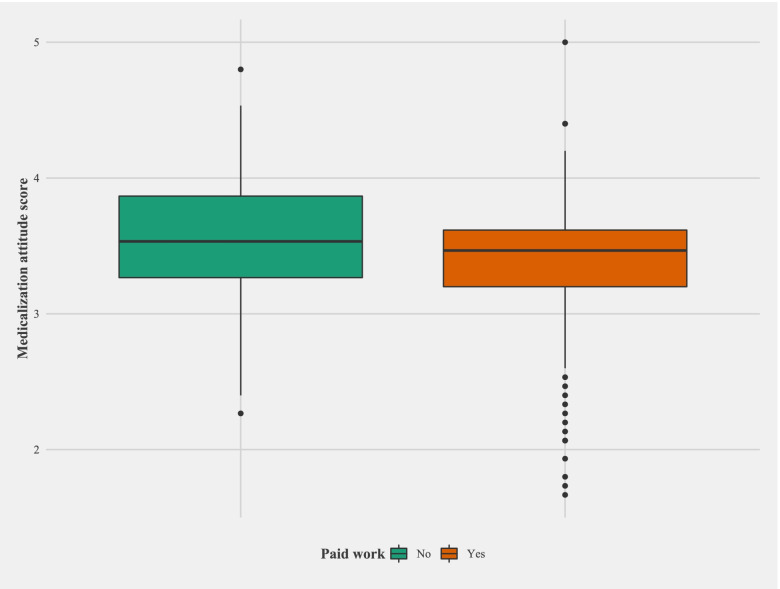
Relationship between having a paid job and attitude towards medicalization in childbirth score

The univariate and multivariate analysis of the variables under study are shown in Table 2. Initially Locus of control (Internality and Chance externality), preparatory and spiritual coping, educational level and having a paid job (*work for which economic remuneration is received, as opposed to work performed in the domestic sphere*) appear to be significantly related to the ATMC score, but when we adjust for all the variables and select the best model (Table 3), only Internal locus (Fig. 3), preparatory coping (Fig. 4) and going out to work (Fig. 5) show an effect on ATMC. Table 3 presents the Final multivariate model of the ATMC and its relationship with other variables after eliminating the sociodemographic variables with lesser contribution to the model. For each point in “Internal locus” and “Preparatory Coping”, the ATMC score was reduced by 0.02 and 0.23 points, respectively, while it increased by 0.18 with not having a paid job.

**Table 2:**
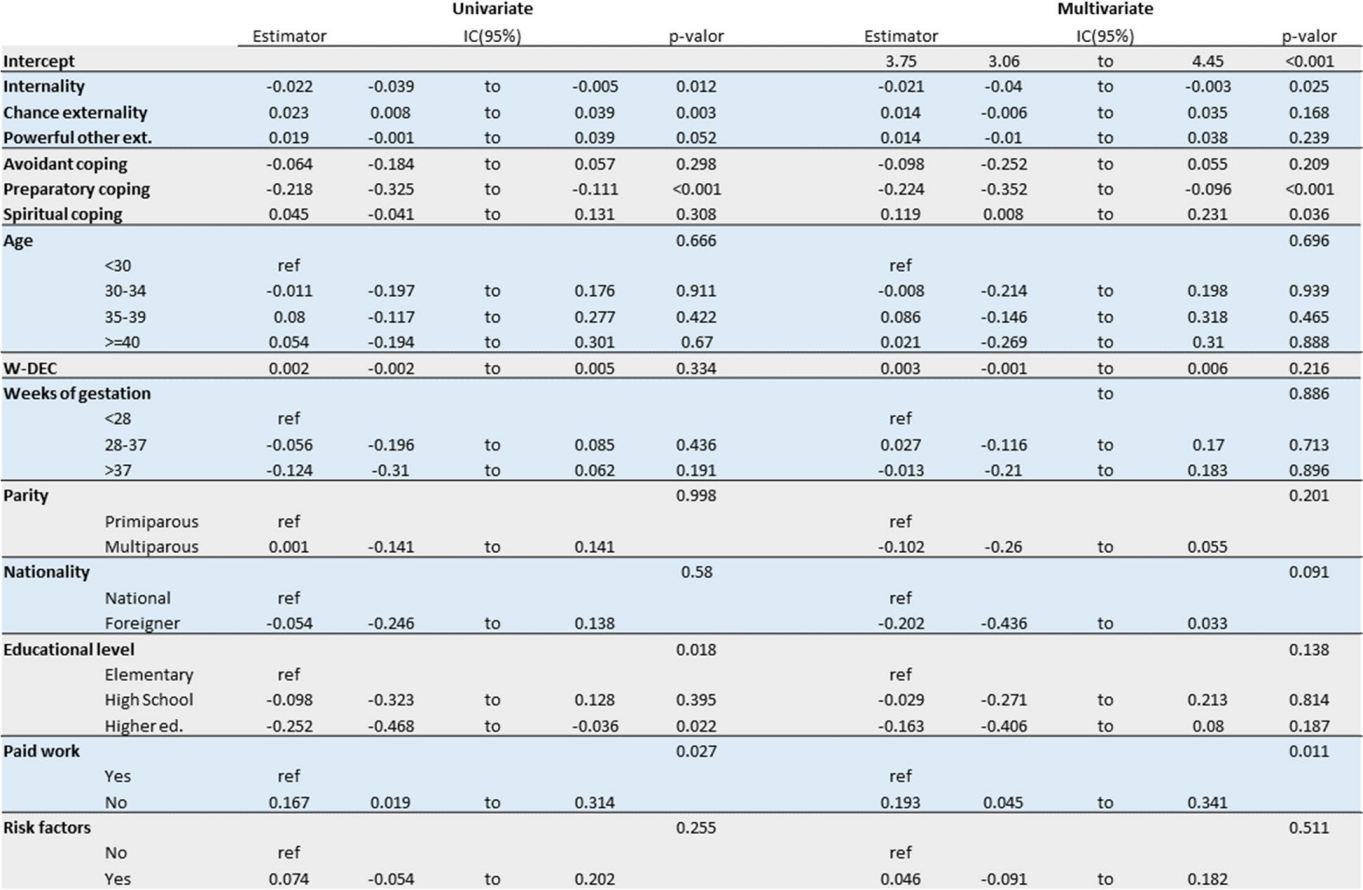
Univariate and multivariate analysis of the attitude towards medicalization and its relationship with other variables

**Table 3:**
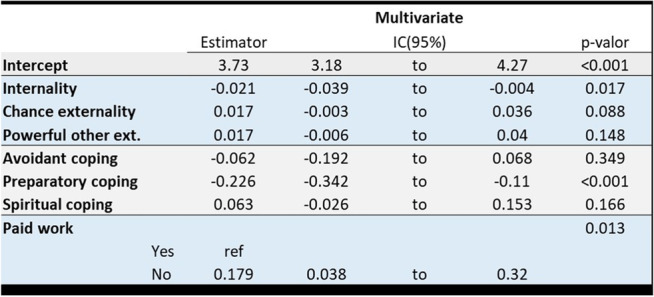
Final multivariate model of attitude towards medicalization and its relationship with other variables

## Discussion

In light of the results of this study, it seems that women in our region prefer a medical delivery. The fact of giving birth in a hospital environment, with the availability of a professional at their disposal, emergency services in case something happens and continuous monitoring of the baby's health seems to provide them with security. Only a minority consider that medical interventions in childbirth could be dangerous or affect the childbirth experience.

Women’s attitudes toward childbirth may be shaped by their backgrounds, experiences, beliefs, values, and needs, which in turn may reflect the social and political environment (in terms of resources) of childbirth and the dominant discourse of childbirth in a culture, or particular country [41]. Nevertheless, every intervention presents the possibility of untoward effects and additional risks that engender the need for more interventions with their own inherent risks. Unintended consequences to intrapartum interventions make it necessary to promote natural childbirth processes and advocate for policies that focus on education, ensuring informed consent and shared decision-making [4]. It is also essential to know what psychological factors influence or are related to having a more favourable or less favourable attitude towards the medicalization of childbirth, not only because of its influence on childbirth choices and satisfaction with the experience [22–24], but also because they give us the possibility of proposing education that is better adapted to the woman’s needs.

The results of the present study show that both the locus of control and the coping strategies normally used to deal with stress, in general, could be related to their attitude towards medicalization in childbirth; specifically, presenting an internal locus of control can be linked to a more unfavourable ATMC, as is adopting preparatory stress coping strategies. Moreover, these relationships are seen independently of all the sociodemographic variables studied, with the exception of work. In this case, not having a paid job is associated with a more favourable attitude toward medicalization, and conversely, working outside is associated with a less favourable attitude toward medicalization. We have not found anything in the literature on this issue, but, perhaps the fact of working outside the home and having to solve the usual problems that arise in any job could somehow “empower” these women and make them feel more prepared to face new challenges, such as a natural childbirth.

Not surprisingly, women with an internal locus of control are less amenable to medicalization. Studies already published have related having an internal locus of control with the desire to actively participate in the childbirth process [33, 42], with the desire for information [31, 42] and with presenting more behaviour of preparation and self-care of health during pregnancy [28, 30]. According to some authors, the health locus of control may be an important pathway through which education, or health literacy, influences health behaviour [43, 44]. The internal locus of control has also been associated with the use of coping strategies focused on the problem, and are more adaptive than those strategies usually used by people with an external locus of control, which are more focused on emotions [45].

Moreover, a recent study concludes that pregnant women who use more adaptive coping strategies, especially planning-preparing coping, reported lower levels of anxiety/depression and engaged more in healthy behaviour. The study also suggests that obstetricians and health care providers should pay more attention to the roles of coping strategies, especially planning-preparation and avoidance coping, in improving health-promotion/health-harming behaviour patterns of pregnant women [46]. In the present study, it is seen that the majority of adopting preparatory coping strategies against stress are related to having a less favourable attitude towards medicalization, which could indicate that preparatory strategies could potentially be good for reducing the stress of future childbirth, making the woman feel more prepared to face it without the need for high technology or medical aid. Given that childbirth is an isolated event, adaptation strategies for dealing with the future situation are therefore be of a variable nature [34, 37] and, therefore, could be susceptible to modification if they turned out not to be useful for dealing with the delivery process.

In view of these results, the interaction between locus of control, coping strategies and attitude towards the medicalization of childbirth could be considered when proposing educational interventions, for example to promote a more natural delivery, ensure informed consent and shared decision making. Identifying the type of locus that the woman presents can be useful for offering the most appropriate strategy. The professional can represent a source of authority and be motivational [47] in the case of women who present an external “powerful others” locus, and therefore work could be done to encourage them to use preparatory coping strategies, if one wants to encourage a less favourable attitude towards medicalization in childbirth. If the woman presents an internal locus of control, the influence of the professional would be less, and the intervention perhaps less necessary. In this case, it might be more appropriate for them to self-manage their own resources. Both the consultation with the midwife and the group sessions of Maternal Education could be an appropriate space to start working on this type of intervention, but more research is needed on the most appropriate type of intervention and its implementation in clinical practice.

## Limitations

As this is an observational study, causality cannot be established with any of the associations. On the other hand, a longitudinal study would have allowed us to observe the changes that occur throughout pregnancy in each of the study variables.

Another limitation could derive from the self-selection of the sample, although measures have been taken to avoid introducing bias: the women were selected by 25 midwives belonging to 28 primary health care centres located in various population centres, both rural and urban, and of different socioeconomic and social characteristics. These 28 centres are part of 6 different Integrated Health Organizations. In light of the sociodemographic data, it can be said that the women in our study are representative of the study population.

## Conclusion

The results of this study suggest that women have a favourable attitude towards medicalization of childbirth. Both the Locus of Control (LC) and the Coping Strategies (CS) of women during pregnancy are related to this attitude. Specifically, having an internal LC and using preparative CS both lower the probability of presenting a favourable attitude towards medicalization, while the lack of a paid job raises the probability. The influence of these psychological factors must be taken into account in the development of content and interventions that promote a more natural birth.

## Data Availability

The data sets generated and/or analysed during the current study are not yet publicly available as they are still being processed by the research team for further publication, but will be made available from the corresponding author upon reasonable request.

## Abbreviations

ATMC: Attitude towards Medicalization of Childbirth
LC: Locus of Control
CS: Coping strategies (stress coping strategies)
SLT: Social Learning Theory
IHO: Integrated Health Organizations
NuPCI: Revised Prenatal Coping Inventory

## References

[1] Benyamini Y, Molcho ML, Dan U, Gozlan M, Preis H (2017). Women’s attitudes towards the medicalization of childbirth and their associations with planned and actual modes of birth. Women Birth.

[2] Ministerio de Sanidad. Atención perinatal en España: Análisis de los recursos físicos, humanos, actividad y calidad de los servicios hospitalarios, 2010-2018 [Publicación en Internet. Ultimo acceso Abril 2022]. Madrid: Ministerio de Sanidad; 2021. NIPO en línea: 133-21-017-X. https://cpage.mpr.gob.es/.

[3] Hammer RP, Burton-Jeangros C (2013). Tensions around risks in pregnancy: a typology of women's experiences of surveillance medicine. Soc Sci Med.

[4] Jansen L, Gibson M, Bowles BC, Leach J (2013). First do no harm: interventions during childbirth. J Perinat Educ.

[5] Foucault M. El nacimiento de la clínica: una arqueología de la mirada médica. Siglo XXI de España Editores, S.A.; Presses Universitariesde France edición (15 diciembre 1999). ISBN-10 843231014X.

[6] Illich I. Nemesis médica: La expropiación de la salud [Internet]. Ed. Barral, 1975 [citado 14 de junio de 2021]. ISBN: 84-211-0330-X. Disponible en: https://dialnet.unirioja.es/servlet/libro?codigo=796115.

[7] Busfield J (2017). The concept of medicalisation reassessed. Sociol Health Illn.

[8] Smith R (2003). Limits to medicine Medical nemesis: the expropriation of health. J Epidemiol Community Health.

[9] Smith R (2002). In search of "non-disease". BMJ.

[10] Kishore J (2013). A Dictionary of Public Health.

[11] de Guillerna RM, Márquez S. La medicalización de la vida y sus protagonistas. Eikasia: revista de filosofía, II. 2007;(8):65-86. ISSN-e 1885-5679. http://www.revistadefilosofia.org/.

[12] Clesse C, Lighezzolo-Alnot J, de Lavergne S, Hamlin S, Scheffler M (2018). The evolution of birth medicalisation: A systematic review. Midwifery.

[13] Conrad P (2005). The shifting engines of medicalization. J Health Soc Behav.

[14] Barker KK (2008). Electronic support groups, patient-consumers, and medicalization: the case of contested illness. J Health Soc Behav.

[15] Béhague DP, Victora CG, Barros FC (2002). Consumer demand for caesarean sections in Brazil: informed decision making, patient choice, or social inequality? A population based birth cohort study linking ethnographic and epidemiological methods. BMJ.

[16] Van der Hulst LA, van Teijlingen ER, Bonsel GJ, Eskes M, Birnie E, Bleker OP (2007). Dutch women's decision-making in pregnancy and labour as seen through the eyes of their midwives. Midwifery.

[17] Kaimal AJ, Kuppermann M (2012). Decision making for primary cesarean delivery: the role of patient and provider preferences. Semin Perinatol.

[18] Jouhki MR, Suominen T, Åstedt-Kurki P (2017). Giving birth on our own terms-Women's experience of childbirth at home. Midwifery.

[19] Downe S, Finlayson K, Oladapo OT, Bonet M, Gülmezoglu AM (2018). What matters to women during childbirth: A systematic qualitative review. PLoS ONE.

[20] Feeley C, Thomson G (2016). Tensions and conflicts in 'choice': Womens' experiences of freebirthing in the UK. Midwifery.

[21] Jackson MK, Schmied V, Dahlen HG (2020). Birthing outside the system: the motivation behind the choice to freebirth or have a homebirth with risk factors in Australia. BMC Pregnancy Childbirth.

[22] Malacrida C, Boulton T (2014). The best laid plans? Women's choices, expectations and experiences in childbirth. Health (London).

[23] Wilson KL, Sirois FM (2010). Birth attendant choice and satisfaction with antenatal care: the role of birth philosophy, relational style, and health self-efficacy. J Reprod Infant Psychol.

[24] Haines HM, Rubertsson C, Pallant JF, Hildingsson I (2012). The influence of women's fear, attitudes and beliefs of childbirth on mode and experience of birth. BMC Pregnancy Childbirth.

[25] Levenson H (1973). Multidimensional locus of control in psychiatric patients. J Consult Clin Psychol.

[26] Wallston KA, Wallston BS, DeVellis R (1978). Development of the Multidimensional Health Locus of Control (MHLC) Scales. Health Educ Monogr.

[27] Mautner D, Peterson B, Cunningham A, Ku B, Scott K, LaNoue M (2017). How Multidimensional Health Locus of Control predicts utilization of emergency and inpatient hospital services. J Health Psychol.

[28] Tinsley BJ, Trupin SR, Owens L, Boyum LA (1993). The significance of women's pregnancy-related locus of control beliefs for adherence to recommended prenatal health regimens and pregnancy outcomes. J Reprod Infant Psychol.

[29] Rongen A, Robroek SJ, Burdorf A (2014). The importance of internal health beliefs for employees’ participation in health promotion programs. Prev Med.

[30] Kordi M, Heravan MB, Asgharipour N, Akhlaghi F, Mazloum SR (2017). Does maternal and fetal health locus of control predict self-care behaviors among women with gestational diabetes?. J Educ Health Promot.

[31] Holroyd LE, Anders S, Robinson JR, Jackson GP (2018). Use of the Multidimensional Health Locus of Control to Predict Information-Seeking Behaviors and Health-Related Needs in Pregnant Women and Caregivers. AMIA Annu Symp Proc.

[32] Tomás-Sábado J, Montes-Hidalgo J (2016). Versión española de la Escala multidimensional de locus de control de la salud en estudiantes de enfermería [Spanish version of the Multidimensional health locus of control scale innursing students]. Enferm Clin.

[33] Pomeranz M, Arbib N, Haddif L, Reissner H, Romem Y, Biron T (2018). “In God we trust” and other factors influencing trial of labor versus Repeat cesarean section. J Matern Fetal Neonatal Med.

[34] Lazarus Richard S, Susan Folkman. Stress, appraisal, and coping. New York: Springer; 2015. ISBN: 0826141919 9780826141910. https://www.worldcat.org/title/stress-appraisal-and-coping/oclc/949922702?referer=di&ht=edition.

[35] Guardino CM, Schetter CD (2014). Coping during pregnancy: a systematic review and recommendations. Health Psychol Rev.

[36] Yali AM, Lobel M (2002). Stress-resistance resources and coping in pregnancy. Anxiety Stress Coping.

[37] Lorén-Guerrero L, Gascón-Catalán A, Romero-Cardiel MA (2018). Adapting the revised prenatal coping inventory (NuPCI) for use in a Spanish population. J Psychosom Obstet Gynaecol.

[38] Paz-Pascual C, Artieta-Pinedo I, Espinosa M, Bully P, ema-Q Group (2020). Development of two instruments for assessing maternity health needs: protocol of a clinimetric study. BMC Pregnancy Childbirth.

[39] Wijma K, Wijma B, Zar M (1998). Psychometric aspects of the W-DEQ; a new questionnaire for the measurement of fear of childbirth. J Psychosom Obstet Gynaecol.

[40] Ortega-Cejas CM, Roldán-Merino J, Lluch-Canut T, Castrillo-Pérez MI, Vicente-Hernández MM, Jimenez-Barragan M, Biurrun-Garrido A, Farres-Tarafa M, Casas I, Cabrera-Jaime S (2021). Reliability and validity study of the Spanish adaptation of the "Wijma Delivery Expectancy/Experience Questionnaire" (W-DEQ-A). PLoS ONE.

[41] Westergren A, Edin K, Lindkvist M, Christianson M (2021). Exploring the medicalisation of childbirth through women's preferences for and use of pain relief. Women Birth.

[42] Heinze SD, Sleigh MJ (2003). Epidural or no epidural anaesthesia: Relationships between beliefs about childbirth and pain control choices. J Reprod Infant Psychol.

[43] Park CL, Cho D, Moore PJ (2018). How does education lead to healthier behaviours? Testing the mediational roles of perceived control, health literacy and social support. Psychol Health.

[44] Mirzania M, Khajavi A, Kharazmi A, Moshki M (2020). Health literacy and quality of life among Iranian pregnant women: The mediating role of health locus of control. Med J Islam Repub Iran.

[45] Wilski M, Brola W, Tomczak M (2019). Health locus of control and mental health in patients with multiple sclerosis: Mediating effect of coping strategies. Res Nurs Health.

[46] Pasha H, Faramarzi M, Chehrazi M, Bakouei F, Gholinia H, Abdollahi S, Shafierizi S (2022). Health-promotion and health-harming behaviours in pregnant women: role of coping strategies, anxiety, and depression. J Obstet Gynaecol.

[47] Delale EA, Novokmet N, Fuchs N, Dolanc I, Mrdjen-Hodžić R, Karelović D, Janković S, Milanović SM, Cameron N, Missoni S (2021). Stress, locus of control, hope and depression as determinants of quality of life of pregnant women: Croatian Islands' Birth Cohort Study (CRIBS). Health Care Women Int.

